# Factors associated with the prescription of vaginal pessaries for pelvic organ prolapse

**DOI:** 10.6061/clinics/2019/e934

**Published:** 2019-09-04

**Authors:** Suelene Albuquerque Coelho, Luiz Gustavo O Brito, Camila Carvalho de Araújo, Luiza Borges Aguiar, Jorge M Haddad, Paulo C Giraldo, Cássia R T Juliato

**Affiliations:** I Departamento de Ginecologia e Obstetricia, Faculdade de Ciencias Medicas, Universidade Estadual de Campinas, Campinas, SP, BR; II Departamento de Ginecologia e Obstetricia, Faculdade de Medicina (FMUSP), Universidade de Sao Paulo, Sao Paulo, SP, BR

**Keywords:** Survey, Pessary, Pelvic Organ Prolapse, Gynecologists, Practice, Knowledge

## Abstract

**OBJECTIVE:**

To identify the factors associated with the prescription of vaginal pessaries (VPs) as a conservative treatment for pelvic organ prolapse (POP).

**METHODS:**

A cross-sectional study was performed during two annual urogynecology and general obstetrics and gynecology meetings in 2017 (São Paulo, SP, Brazil). A 19-item deidentified questionnaire regarding experiences and practices in prescribing VPs for POP patients was distributed among gynecologists. Our primary outcome was the frequency of prescribing VPs as a conservative treatment for POP. The reasons for prescribing or not prescribing VPs were also investigated. Univariate and multivariate analyses with crude and adjusted odds ratios (ORs) were performed for variables associated with the prescription of pessaries.

**RESULTS:**

Three hundred forty completed surveys were analyzed. Half of the respondents (53.53%) were between 30-49 years old; most of them were female (73.53%), were from the Southeast Region (64.12%), were trained in obstetrics and gynecology (80.24%) or urogynecology (61.18%) and worked in private offices (63.42%). More than one-third (36.48%) attended four or more POP cases/week, and 97.65% (n=332) had heard or knew about VPs for POP; however, only 47.06% (n=160) prescribed or offered this treatment to patients. According to the multivariate analysis, physicians aged 18-35 years (OR=1.97[1.00-3.91]; *p*=0.04), those who participated in a previous urogynecology fellowship (OR=2.34[1.34-4.09]; *p*<0.01), those with relatively high volumes of POP cases (4 or +) (OR=2.23[1.21-4.47]; *p*=0.01) and those with PhD degrees (OR=2.75[1.01-7.54]; *p*=0.05) prescribed more pessaries.

**CONCLUSIONS:**

Most gynecologists did not prescribe VPs. Younger physician age, participation in a previous urogynecology fellowship, a PhD degree, and a relatively high volume of POP cases were associated with increased VP prescription rates.

## INTRODUCTION

Pelvic organ prolapse (POP) is a common and increasingly prevalent disease in adult and elderly women 1. Surgical treatment is the definitive treatment for this disease; however, for patients who do not want to undergo surgery (or have a high anesthetic risk), life-style adjustment, physical therapy and the use of vaginal pessaries (VPs) are possibilities that should be offered as initial treatment options 2. Among the three latter conservative treatments, VPs provide high subjective cure rates (60-80%) 3. Moreover, a systematic review demonstrated that VPs produced a positive effect on the quality of life women 4.

However, there are controversies among physicians regarding the appropriate use of pessaries 5. There are also regional differences in the practice of prescribing VPs; a published survey performed by members of the International Urogynecological Association (IUGA) showed that South America was ranked fourth regarding the consistent or frequent offering of VPs according to 49% of the respondents 6. This value was considered low when compared to North America (87.5%), and to our knowledge, this is only survey to evaluate pessary prescription in South America. In Europe, a nationwide survey in the Netherlands found that 69% of gynecologists with special interest in urogynecology proposed pessary treatment for their patients, and 13% had a written protocol for VP prescription in their department 7. Unfortunately, in Brazil, the public health system does not provide VPs for patients, and we do not have information about whether women are counseled about conservative options. Thus, ignorance about the prescribing patterns of VPs in Brazil by physicians has contributed to the suboptimal education of patients regarding their options for treating POP, and physicians may not provide the best option for all kinds of clinical scenarios. It is important to understand the factors associated with VP prescription so that this treatment may be considered an option by health care providers.

## METHODS

This was a cross-sectional study (survey) performed during two meetings (Jornada de Uroginecologia da FMUSP – April 2017; Congresso da Associação de Obstetrícia e Ginecologia do Estado de São Paulo – August 2017). As physicians were visiting the exhibition area, they were invited to participate in the survey. Medical residents and attending physicians were eligible to participate. The Institutional Review Board of the University of Campinas approved this survey (CAAE 61607416.0.0000.5404).

After explaining the aims of the survey, the participants read and signed an informed consent form. Then, a 19-item, deidentified questionnaire (to reduce attrition and observer bias) about practices and knowledge of pessary management was provided to them. One hundred respondents from the first meeting participated, and 240 respondents from the second meeting responded. This questionnaire was developed specifically for this survey, and although it was not previously pilot tested, it was elaborated by a panel of two coauthors; one of them was an expert in urogynecology (C.R.T. J) and the other was a physiotherapist with a specialization in pelvic floor dysfunctions (S.A.C). The primary outcome was whether the physician offered or prescribed a VP as a treatment option for patients (yes/no). The dependent variables were the characteristics of the gynecologists (age, sex, educational level), settings where they attended to patients, reasons for not prescribing or indicating VPs, management of VPs and complications associated with VPs. We asked the participants to reply the questions as if considering a patient without a previous pessary fitting trial.

A sample size calculation was performed. Previous studies found a prevalence of VP prescription of 60-80% (5,7); considering an absolute estimate of 10% and a significance level of 5%, the minimum sample size was between 81 and 92 patients. Descriptive data were analyzed using SAS version 9.2 (SAS Institute, Cary, NC, USA). The frequencies and percentages of respondents were calculated. Chi-square tests were performed to analyze the association between the dichotomous variables and Student's t-tests were performed for the continuous variables. Chi-square tests for trends were performed to determine whether there were any correlations between the number of attended POP cases or educational level with prescribing or not prescribing VPs. A univariate analysis to calculate a crude odds ratio (OR) was performed if the *p*-value was <0.05; a multivariate analysis was performed for all the variables with a *p*-value <0.15. Missing data was not supplemented with any imputation methods. The significance level was stipulated as 5%.

## RESULTS

Three hundred forty physicians completed the survey. The mean age of the interviewed gynecologists was 40.49±11.44 years (20-70), and most of the physicians were women (73.53%) from the Southeast Region (64.12%) of Brazil. Regarding the education level, 250 (73.52%) physicians had concluded a medical residency in obstetrics and gynecology, and 60.88% had participated in a clinical fellowship in urogynecology. The most frequent types of departments that physicians were affiliated were private offices (63.42%) and public hospitals (45.43%). More than 90% (n=318) reported that they attended POP cases, with a frequency of four or more patients per week (36.28%).

Almost the entire sample (n=331; 97.35%) answered that they knew or heard about VPs (Table 1); however, when they were asked if they prescribed or offered the use of VPs for patients, 52.94% said that they did not. The most frequent reason for not prescribing VPs was that they did not have experience (90.56%). On the other hand, most of the VP prescribers said they often offer the device to patients (57.84%); a ring pessary was the most frequently prescribed type (54.19%), followed by a donut pessary (48.39%). The most frequent reasons for prescribing VPs were that women had no surgical conditions (89.03%), followed by advanced age (68.39%). The mean age of the group of VP prescribers (39.50±10.94) was almost two years younger than the mean age of the VP nonprescribers (41.42±11.84), with no significant difference (*p*=0.12). Sex (*p*=0.12), region (*p*=0.83) and education level (*p*=0.34) were not associated with prescribing/offering VPs to patients.

Most of the physicians scheduled patients for monthly follow-ups for VP cleaning and management (70.32%), and replacement of the device usually occurred when the patient requested it (41.18%) or after one year of use (26.80%). However, most gynecologists counseled women on to clean their pessaries at home (89.68%). More than 80% of the physicians advocated the use of vaginal estrogen with a VP. With regard to complications with the use of VPs, more than half of the prescribers (54.84%) said they observed some complications such as increased vaginal discharge (69.41%) or malodorous discharge (35.29%). Finally, the majority (73.55%) indicated that a VP is a good alternative for POP treatment.

Table 2 presents the univariate and multivariate analysis of the risk factors. Physicians between the ages of 18 and 35 years (OR=1.81[1.03-3.21]; *p*=0.04), those who participated in a previous urogynecology fellowship (OR=3.97[2.48-6.34]; *p*<0.01), those who attended POP patients (OR=8.58[2.53-29.08]; *p*<0.01), and those with a high volume of cases (OR=3.75[2.14-6.59]; *p*<0.01) were more likely to prescribe VPs than those without such experience. After adjusting for confounding factors, all the variables remained significantly associated with the prescription of VPs in the final model (except the variable attending POP patients); relatively young physicians were twice as likely to prescribe VP (OR=1.97[1.00-3.91); *p*=0.01), as well as physicians with a relatively high number of prolapse cases (4+) (OR=2.32[1.21-4.47); *p*=0.01) and those who participated in a previous urogynecology fellowship (OR=2.34[1.34-4.09]; *p*<0.01). Interestingly, physicians with a PhD degree were also associated with increased odds of prescribing VPs in the multivariate analysis (OR=1.75[1.01-7.54]; *p*=0.05)

## DISCUSSION

We found that most of the gynecologists, despite knowing the device and its use for POP treatment, did not offer or prescribe it to their patients. Moreover, young professionals, those with advanced degrees (PhD), those with previous urogynecology fellowship experience and those who attended high POP volumes were associated with VP prescriptions. Regarding the prevalence of VP prescription, the results were different from those in North American and European countries, where the majority of physicians usually prescribe this device. This is reason for concern because VPs are minimally invasive devices that can be feasibly used in any region regardless of its socioeconomic development 8. Despite the considerable number of trained urogynecologists in this sample, we still observed that majority did not prescribe VPs.

There are some hypotheses about this low to moderate frequency of VP prescription. One hypothesis is the lack of training on inserting VPs during residency. Most of the nonprescribers reported that they did not have experience administering VPs for their patients. Perhaps physicians do not know how to measure the vaginal cavity to identify the appropriate pessary for each patient. We do not have published studies about this topic in Brazil; however, a study in the US found that obtaining experience in pessary fitting and formal pessary-specific didactics and working with advanced practitioners improved residents' confidence in pessary use 9. Another possibility is the involvement of a multidisciplinary team. In our service, physiotherapists work with urogynecologists and nurses to provide assistance to women using VPs. In the US, an e-mail survey of three professional nursing organizations found that 86.4% of the nurses managed pessaries in the practice setting 10; however, in the UK, doctors were significantly more involved in pessary care than nurses or physiotherapists 11. This could be a feasible and cost-effective option for any health care unit, sharing the responsibilities of care for these patients.

Most of the reasons that were noted as indications for VP use were similar to that available in the literature, such as patients who have contraindications for surgery, regardless of the type of VP that is chosen 7,11,12. The most frequent devices were the ring and donut pessaries, similar to other studies 7,11,12. An American study found that gynecologists preferred the ring because it was the easiest to use 13. It is still unknown whether one device is superior to another; prospective, randomized studies are necessary to address this question.

With regard to follow-up for the cleaning and management of VPs, most gynecologists scheduled a 4-week follow-up appointment. In the Netherlands, the first follow-up visit generally occurred between 6 and 12 weeks 7. There is no evidence in the literature about the ideal or minimal follow-up intervals after initial VP placement. Moreover, we do not know the proportion of patients who are able to learn to clean and replace the pessary themselves. Our local experience gives us the impression that patients prefer to return for a follow-up visit to have their VPs cleaned and reinserted; however, a prospective study regarding this outcome is needed to confirm this statement.

Regarding replacing the VP, most of the gynecologists performed replacement after one year of use or when the patient requested it. This is probably related to the fear of complications. We do not have a standardized recommendation about whether to replace the device or not and when should we do so. Among physicians who prescribe VPs, increased vaginal discharge was the most common complication reported, similar to results published in the literature 14.

To our knowledge, this is the first study about pessary use practices for POP considering gynecologists in a Latin American country, such as Brazil. Moreover, we believe that the external validity was not impaired because most of our sampling was conducted at a general obstetrics and gynecology meeting, accurately representing reality. However, there were limitations; they were mostly related to survey biases (response bias, social desirability bias) and possible selection bias (most of the participants participated in a clinical fellowship in urogynecology), meaning that the frequency may be lower than the value we found among the general practitioners. However, we believe these results will have good external validity because it is hypothesized that practitioners who attend meetings have more knowledge about medical treatments regardless of their specialty. Thus, these physicians are probably updated on the medical literature.

In conclusion, there was a low rate of VP prescription despite the majority of the doctors knowing about the device and its use for POP because there was a lack of experience in VP use. The results of this study emphasize the importance of training and education among gynecologists, mainly during residency, about other POP treatment options; this will empower patients with knowledge regarding all their possibilities, allowing them to choose the best treatment for their particular situation. A feasible and more complete scenario would be working with a multidisciplinary team, where physicians along with nurses and/or physiotherapists could share the task of identifying these patients.

## AUTHOR CONTRIBUTIONS

Coelho SA was responsible for the project development, data collection, data analysis, and manuscript editing. Brito LGO, Haddad JM, Giraldo PC and Juliato CRT were responsible for the project development, data analysis, and manuscript writing. de Araújo CC was responsible for project development and data collection. Aguiar LB was responsible for the data collection and analysis.

## Figures and Tables

**Table 1:** Practice and knowledge regarding the prescription of pessaries among the surveyed gynecologists.

Variables	Frequency (%)	Variables	Frequency (%)
**Do you know (or have you heard) about the pessary?**		How often do you schedule patients for follow-up for cleaning and management of the pessary*?	
Yes	332 (97.65)	Every month	109 (70.32)
No	8 (2.35)	Every two months	14 (9.03)
**Do you prescribe (or offer the use) the pessary for patients?**		Every three months	25 (16.13)
Yes	160 (47.06)	6 months or more	11 (7.10)
No	180 (52.94)	How often do you replace the pessary for another*?	
If no, why not?*		After one year of use	41 (26.80)
Does not know about the pessary	9 (5.00)	After two years of use	34 (22.22)
Does not have enough experience	163 (90.56)	After three (or more) years of use	17 (11.11)
Does not like prescribing it	5 (2.78)	When the patient requests	63 (41.18)
Has not had good results with it	4 (2.22)	**Do you counsel women to clean their pessaries at home?**	
If yes, how often do you prescribe it*		Yes	139 (89.68)
Always	37 (36.27)	No	16 (10.32)
Sometimes	59 (57.84)	**Do you prescribe vaginal estrogen associated with the use of pessary?**	
Only when patients ask about treatments other than surgery	6 (5.89)	Yes	130 (83.87)
Type of pessary model that you usually prescribe*:		No	25 (16.13)
Ring	84 (54.19)	**In your daily practice, do you notice patient complications when using pessaries?**	
Donut	75 (48.39)	Yes	85 (54.84)
Ring with support	31 (20.00)	No	70 (45.16)
Cube	9 (5.81)	If yes, what were the main complications that you have diagnosed in these women*?	
Gelhorn	4 (2.58)	Increased vaginal discharge	59 (69.41)
Others	2 (1.29)	Bacterial vaginosis/ Candidiasis	23 (27.06)
What are your main indications for the use of a pessary*?		Malodorous discharge	30 (35.29)
Women with no surgical conditions	138 (89.03)	Ulceration	29 (34.12)
Older women	106 (68.39)	Bleeding	19 (22.35)
Apical prolapse	53 (34.19)	In general, do you think that the pessary use is a good alternative to POP treatment*?	
Anterior prolapse	36 (23.23)	Yes	115 (74.19)
Posterior prolapse	20 (12.90)	Sometimes	20 (12.90)
Urinary retention	11 (7.10)	With a good rationale/indication	21 (13.55)

* More than one answer was possible.

**Table 2:** Risk factors associated with pessary prescription for pelvic organ prolapse.

Variables	Pessary prescription (%)		Crude OR (95%CI); *p*-value	Adjusted OR (95%CI); *p*-value
	**Yes**	**No**		
**Age (years)**			1.32 (0.99-1.74); 0.05	1.34 (0.97-1.87)
18-35	74 (52.86)	66 (47.14)	1.81 (1.03-3.21); 0.04	1.97 (1.00-3.91); 0.04
36-50	62 (50)	62 (50)	1.62 (0.91-2.90); 0.10	1.59 (0.83-3.06); 0.16
50+	29 (38.16)	47 (61.84)	Ref	Ref
**Sex**			0.68 (0.41-1.10); 0.12	0.85 (0.48-1.50); 0.58
Female	115 (46)	135 (54)		
Male	50 (55.55)	40 (44.44)		
**Brazilian region**			1.07 (0.086-1.35); 0.52	-------------------
North	2 (33.33)	4 (66.67)	Ref	Ref
Northeast	29 (47.54)	32 (52.46)	1.81 (0.30-10.64); 0.51	
Midwest	10 (52.63)	9 (47.37)	2.22 (0.35-15.18); 0.42	
Southeast	104 (47.71)	114 (52.29)	1.82 (0.33-10.17); 0.49	
**South**	20 (55.56)	16 (44.44)	2.5 (0.40-15.43); 0.32	
Education level			1.27 (0.95-1.70); 0.10	
Medical residency	115 (46)	135 (54)	Ref	Ref
Master’s degree (e.g., MsC, MPH)	29 (51.79)	27 (48.21)	1.26 (0.71-2.25); 0.43	1.04 (0.53-2.03); 0.93
Doctorate degree (e.g., PhD, DsC)	15 (65.22)	8 (34.78)	2.20 (0.90-5.38); 0.08	2.75 (1.01-7.54); 0.0.05
Postdoctorate research fellow	6 (54.54)	5 (45.46)	1.41 (0.42-4.74); 0.58	0.63 (0.11-5.51); 0.50
Type of department where the physician works*				
Private office	109 (50.70)	106 (49.30)	1.24 (0.80-1.94); 0.32	
Private hospital	66 (51.97)	61 (48.03)	1.23 (0.79-1.91); 0.35	
Public hospital	81 (52.60)	73 (47.40)	1.33 (0.86-2.04); 0.18	
Academic hospital	66 (54.10)	56 (45.90)	1.40 (0.90-2.19); 0.15	
**Clinical fellowship in urogynecology**			3.97 (2.48-6.34); <0.01	2.34 (1.34-4.09); <0.01
Yes	127 (61.35)	80 (38.65)		
No	38 (28.57)	95 (71.43)		
**Do you attend clinical cases of pelvic organ prolapse?**			8.58 (2.53-29.08); <0.01	0.69 (0.10-4.54); 0.70
Yes	162 (51.76)	151 (48.24)		
No	3 (11.11)	24 (88.89)		
**How many cases of genital prolapse do you attend per week?**			1.53 (1.28-1.83); <0.01	1.29 (1.05-1.59); 0.02
1	38 (36.89)	65 (63.11)	Ref	Ref
2	31 (46.97)	35 (53.03)	1.52 (0.81-2.84); 0.19	1.11 (0.56-2.19); 0.76
3	16 (48.48)	17 (51.52)	1.61 (0.73-3.55); 0.24	1.14 (0.49-2.65); 0.76
4+	79 (68.70)	36 (31.30)	3.75 (2.14-6.59); <0.01	2.32 (1.21-4.47); 0.01

* Dummy variables.
